# Outpatient Antibiotic Use and Prevalence of Antibiotic-Resistant Pneumococci in France and Germany: A Sociocultural Perspective

**DOI:** 10.3201/eid0812.010533

**Published:** 2002-12

**Authors:** Stephan Harbarth, Werner Albrich, Christian Brun-Buisson

**Affiliations:** * University of Geneva Hospitals, Geneva, Switzerland; † Beth Israel Deaconess Medical Center, Boston, Massachusetts, USA; ‡ University Hospital Henri Mondor, Créteil, France

**Keywords:** Antibiotics, therapeutic use, culture, drug resistance, France, Germany, physician’s practice patterns, epidemiology, Streptococcus pneumoniae

## Abstract

The prevalence of penicillin-nonsusceptible pneumococci is sharply divided between France (43%) and Germany (7%). These differences may be explained on different levels: antibiotic- prescribing practices for respiratory tract infections; patient-demand factors and health-belief differences; social determinants, including differing child-care practices; and differences in regulatory practices. Understanding these determinants is crucial for the success of possible interventions. Finally, we emphasize the overarching importance of a sociocultural approach to preventing antibiotic resistance in the community.

The epidemiology of antibiotic-resistant *Streptococcus pneumoniae* varies tremendously between different countries and continents (1). In Europe, high rates of penicillin-resistant pneumococci have been recorded in France and Spain, whereas countries like Germany and Switzerland are only marginally affected (2,3). The reasons for the uneven geographic distribution of antibiotic-resistant pneumococci are not fully understood.

In this article, we focus on a comparison of pneumococcal resistance rates between Germany and France, two neighboring European countries with well-developed health-care systems accessible for virtually the entire population. Moreover, living standards, expenditures on health, and key survival statistics (infant deaths, life expectancy) are roughly equivalent, which allowed us to assume that at least in terms of general health indicators both countries could be judged to be comparable (4). We reviewed recent epidemiologic data about antibiotic resistance in clinically relevant pneumococcal isolates of patients in Germany and France and explored different hypotheses to explain the observed differences between the two countries. The main questions addressed are: 1) Do important differences exist in antibiotic-prescribing practices in the outpatient setting? 2) Do the factors influencing decisions on antibiotic use differ? 3) Are these differences related to sociocultural and other macro-level determinants? In particular, we sought to offer potential methods for future international comparisons designed to aid in developing effective strategies for decreasing the spread of antibiotic-resistant microorganisms in the community.

## Methods

A computer-based literature review was undertaken with the MEDLINE database from 1980 to the present. While references were sought by using specific subject headings related to differences in the prevalence of antibiotic-resistant pneumococci between Germany and France and reasons for the observed disparity (e.g., antibiotic use and prescribing), the paucity of relevant retrievals prompted us to repeat the search by using keywords specific for each of the questions asked. This extended search included articles about differences in economic and sociocultural determinants (e.g., perception of illness, societal background of pharmaceutical consumption). Additional references were identified from the references cited in these reports and personal files. Papers in English, German, and French were reviewed. Antibiotic use on a national level was expressed as defined daily doses (DDD) of different antibiotic agents per 1,000 inhabitants per day, one DDD being the standard daily dose of an antibiotic agent for 1 day’s treatment (5).

### Epidemiology of Resistant Pneumococci

**Figure 1:**
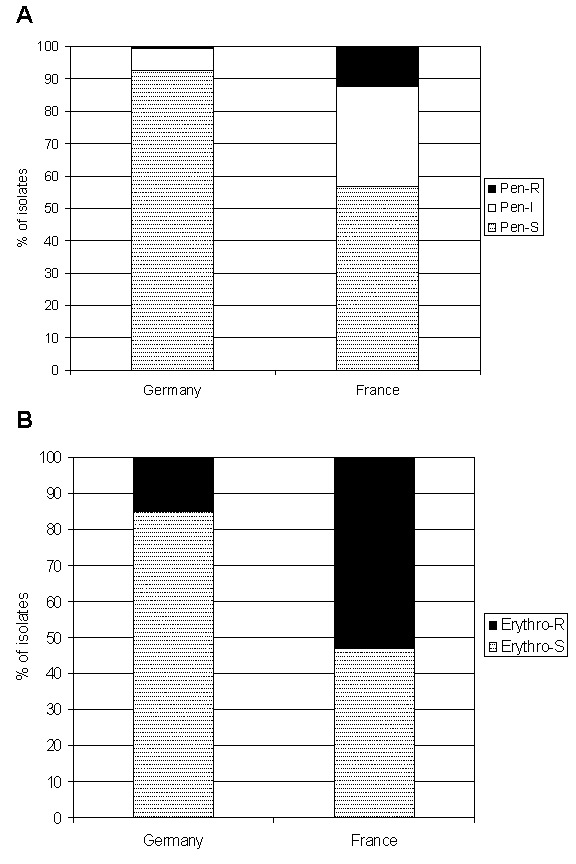
Summary of published aggregate data on the prevalence of pneumococci with intermediate (MIC >0.12 mg/L) and high-level (MIC >2.0 mg/L) resistance (A), and the prevalence of erythromycin-resistant pneumococci (B), France and Germany (3,6,7,9–11).

Among all clinical isolates of *S. pneumoniae* collected from patients of all ages throughout Europe in 1998, 93% (n=168) were susceptible to penicillin (MIC <0.06 mg/L) in Germany, whereas only 47% of French isolates (n=167) remained fully penicillin-susceptible (6). In the same multinational study, 4% and 47% of pneumococcal isolates were erythromycin-resistant (MIC >1 mg/L) in Germany and France, respectively. A national surveillance study about the prevalence of penicillin-resistant *S. pneumoniae* recovered from patients with respiratory tract infections in Germany from 1998 to 1999 showed that of 961 isolates, 93% were fully susceptible to penicillin G and 6% had intermediate susceptibility (7). Three strains expressed high-level resistance to penicillin (MIC >2 mg/L) in that study. In contrast, several recent reports confirm the high prevalence of antibiotic-resistant pneumococci in France (2,3,8–10). For instance, a national surveillance study conducted in France in 1999 demonstrated that the prevalence of penicillin-nonsusceptible (MIC >0.12 mg/L) and erythromycin-resistant pneumococcal isolates (n=14,178) were 43% and 51%, respectively (10). We found that the prevalence of antibiotic-resistant pneumococci is sharply divided between France and Germany. Figure 1 (A and B) summarizes currently available aggregate data on the prevalence of penicillin- and erythromycin-resistant pneumococci in clinical isolates from both countries (3,6,7,9–11).

### Explanatory Dimensions

**Figure 2:**
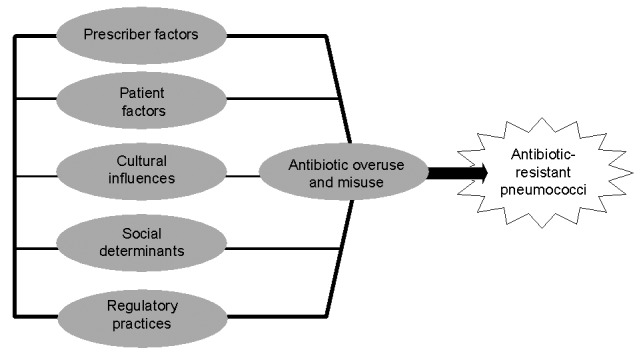
Framework of nonmicrobiologic factors influencing outpatient antibiotic use and prevalence of pneumococcal resistance.

To explain the differences in pneumococcal resistance rates, we identified several dimensions that influence decisions on antibiotic use. These dimensions are derived from the concept that outpatient antibiotic use not only depends on clinical and microbiologic considerations and the frequency of respiratory tract infections but is also related to sociocultural and economic factors (12–14). More precisely, the first dimension of our proposed framework (Figure 2) concerns the prescribers of antibiotics, physicians, who may differ in their use, dosing, and choice of antibiotic agents. The second dimension concerns patient demand and health-belief differences. A third group of determinants of antibiotic consumption is linked to macro-level factors influencing the prescription of antibiotics, such as sociocultural factors (e.g., child-care practices) and regulatory health-care policies. This article has been structured along these explanatory dimensions. Finally, we discuss competing explanations and implications of the presented data.

### Volume of Outpatient Antibiotic Use

The association between community use of antibiotics and antibiotic-resistant pneumococci has been amply demonstrated (15–17). This relationship raises the question of whether differences in the volume and pattern of outpatient antibiotic use exist between Germany and France.

**Figure 3:**
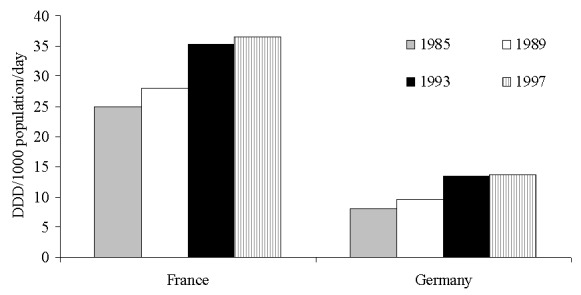
Outpatient antibiotic utilization (18,19), France and Germany, 1985–1997. DDD, daily defined doses.

Analyses of national sales data from Germany and France are summarized in Figure 3 (18,19). These data show that, from 1985 to 1997, retail sales of oral antibiotics in France were almost three times higher than sales in Germany. For instance, in 1997, France used 36.5 DDD/1,000 population/day versus 13.6 DDD/1,000 population/day in Germany (Figure 3) (19). In addition, Germany had a higher relative use of narrow-spectrum penicillins, cotrimoxazole, and tetracyclines and a much lower use of broad-spectrum penicillins, cephalosporins, and fluoroquinolones, compared to France (2,19). Overall, among 18 industrialized countries, Germany had the third lowest and France had the highest antibiotic utilization rate in the outpatient setting throughout the 1990s (18,19).

### Antibiotic-Prescribing Practices for Respiratory Tract Infections

Antibiotic-prescribing practices for respiratory tract infections vary tremendously in France and Germany. Antibiotic prescription rates in France and Germany for common cold and tonsillopharyngitis were 48.7 and 94.6 versus 7.7 and 69.6 per 100 outpatient consultations, respectively (Table) (12). A French survey showed that, during a 3-month period in 1991, 25% of the French population was treated with an antibiotic, compared with 17% in 1980 (22). In particular, the frequency of respiratory tract infections with a presumed viral cause that were diagnosed and treated with antibiotics increased by 86% for adults and by 115% for children in the 11-year period (22).

**Table:** Comparison of patterns of antibiotic prescribing and diagnostic tests for respiratory tract infections, France and Germany^a^

Variables	France	Germany
Average no. office visits for acute tonsillopharyngitis per 1,000 population	136	51
Average no. antibiotic prescriptions per 100 office visits for acute tonsillopharyngitis	94.6	69.6
Average no. office visits for common cold per 1,000 population	253	19
Average no. antibiotic prescriptions per 100 office visits for common cold	48.7	7.7
% of patients not receiving antibiotics at first office visit for		
Suspected community-acquired pneumonia	8	23
Acute bronchitis	7	31
Exacerbation of chronic bronchitis	5	26
Viral lower respiratory tract infection	20	41
% of cases of suspected lower respiratory tract infection with diagnostic tests performed		
Chest radiograph	18	27
Peripheral blood leukocyte count	14	27
Microbiologic sputum examination	3	12

^a^Sources: (12,20,21).

A pan-European survey showed marked differences in the rate of nonprescription of antibiotics at the first consultation for respiratory tract infections (Table) (20). In Germany, the absence of antibiotic prescription reached 41%, even in a case of a suspected pneumonia (21). This lower rate of prescriptions can be explained by a higher recourse to diagnostic investigations and a watchful waiting approach in Germany (21). In fact, in 43% of all suspected cases of respiratory tract infection in Germany, diagnostic tests were performed, compared to 21% in France (Table). Another recently published study (23) confirmed that lower respiratory tract infections seen by general practitioners in France led to few requests for supplementary investigations. Thus, to decrease diagnostic uncertainty and inappropriate prescribing for acute bronchitis and mild exacerbation of chronic bronchitis in France, more precise diagnostic criteria and cost-effective tests are needed.

A study by Guillemot et al. (24) also demonstrated that frequent low-level prescribing of penicillin selects for resistant strains of *S. pneumoniae* in the oropharyngeal flora. When finally prescribed, penicillin agents are usually given in higher doses to German patients than French patients. In a European study, 30% of German adult patients received >3g per day of amoxicillin for lower respiratory tract infections, whereas French patients received considerably lower antibiotic dosages (21). In another survey from France, a high percentage of antibiotic prescriptions were underdosed as compared to clinical recommendations, particularly in children (25). Moreover, some authorities have linked the high prevalence of penicillin-nonsusceptible pneumococci in France to widespread replacement of amino-penicillins by oral cephalosporins, many of which achieve a T > MIC (time for which non–protein-bound concentrations exceed the MIC) of <40% for *S. pneumoniae,* resulting in inadequate killing of bacteria (26). In contrast, the prescription of high-dose amino-penicillins in Germany may be an additional factor contributing to the lower prevalence of penicillin-resistant pneumococci in that country (27).

### The Cultural Perspective

Cultural factors determine which signs and symptoms are perceived as abnormal and thus require medical care and pharmaceutical treatment. Illness perception influences help-seeking behavior and clinical outcome (28). In particular, cultural views of infectious conditions that require antibiotic treatment differ between countries (14).

Many French people seeking medical care because of cough and sputum production request to be treated by antibiotics; by contrast, most Germans consider such treatment as unnecessary overmedication (Table) (12,29). In Germany, many patients accept individualized, complementary medicine and its most refined form, homeopathy, as an equivalent approach for the treatment of respiratory diseases, since great attention is given to improving the body’s natural defense (30,31). A survey commissioned by the European Union among 1,577 opinion leaders in the health-care sector showed that alternative medicines such as homeopathy were supported by 42% of survey participants in Germany versus 23% in France (32). A recently published survey among 2,111 Germans >16 years of age showed that 83% had some sympathy for complementary medicine, whereas 40% disliked antibiotics because they could undermine natural immunity (33). Another opinion poll among 2,647 Germans indicated that the prevalence of using alternative medicine in Germany was the highest among all industrialized countries: 65% in 1996; in 1970, the corresponding figure was 52% (34). Most participants (84%) seemed motivated to use alternative methods largely because of strong misgivings about the potential adverse effects of pharmacotherapy (34).

Based on these health-belief factors, most German physicians follow a less aggressive, watchful-waiting approach in the case of non–life-threatening infections. In particular, German physicians agree that antibiotics are not first-line drugs for the treatment of uncomplicated respiratory tract infections. Indicative of the general attitude is this statement by a German general practitioner: “We never give antibiotics for a common cold ….On the first visit we would only give aspirin. After five days we would do a blood sedimentation and listen to the lungs. Then we might give antibiotics” (35).

In France, physicians have repeatedly reported that unrealistic patient expectations, patient pressure to prescribe antibiotics, and insufficient time to educate patients about the inefficacy of antibiotics for upper respiratory tract infections are the major reasons why antibiotics are prescribed for these self-limiting diseases (12,29,36,37). In a Pan-European survey (36), the demand index for antibiotics among patients in France was 2.2, surpassed only by Turkey (2.4). In that survey, France was the only European country where >50% of the interviewees definitely expected an antibiotic for the treatment of “flu.” Most notably, 82% of French mothers expected antibiotics for their child’s earache (36). In another recently published survey, French parents agreed more strongly than physicians that “all ear infections should be treated with antibiotics” (38).

However, French physicians may overestimate the extent to which patient satisfaction depends only on receiving an antibiotic prescription; therefore practitioners should be convinced that the primary determinant of patient satisfaction is not prescribing antibiotics but rather, effective communication about the patient’s illness (39). For instance, in a recently published study from Nottingham, United Kingdom, antibiotic use for acute bronchitis was reduced by 25% in those patients who received information and reassurance about the benign nature of their disease (40).

### The Social Perspective

Social factors also influence antibiotic use and resistance rates in France and Germany. This influence can be best illustrated by otitis media, the leading reason for excessive antimicrobial use in French children (20). Attendance at a child-care center outside the home correlates strongly with an increased risk of otitis media and acquisition of drug-resistant pneumococci (16,41). Therefore, the great differences in the availability and usage of nonparental day-care facilities between France and Germany are not unexpected.

In France, a long tradition of early childhood education exists in the public sector. Known as “écoles maternelles,” nearly 100% of 3- to 5-year-olds attend these publicly funded pre-schools; about 35% of 2-year-olds also attend (42). In contrast, <10% of German infants in this age group were in the care of an external child-care provider (43). In 1998, 340 nursery places per 100,000 population were available in France, compared to 200 places per 100,000 population in Germany (43). Thus, many more French infants are in the care of an external child-care provider. If they enter child-care, German children enter it later than French children; this practice delays the peak incidence and the cumulative burden of otitis media and associated antibiotic use (44).

Because of the transmission of antibiotic-resistant pneumococci among infants in nurseries in France, a panel of national experts recommended encouraging alternatives that could delay placement in day-care centers until children are 18 months old (45). However, this recommendation seems difficult to follow, since attendance at a child-care center outside the home is a necessity for many families. In fact, France has a high proportion of women employed outside the home: in 1990, 72% of the women ages 25 to 54 years were employed in France compared to 60% in Germany (46). Moreover, <40% of single mothers in Germany are employed, compared to 82% in France (47). Since out-of-home child-care practices are unlikely to change and the proportion of two-career families will likely not decrease in France, promoting smaller child-care size, grouping children in small sub-units, and providing pneumococcal vaccinations could possibly reduce the risk of pneumococcal cross-infection (48–50).

By contrast with Germany, another important risk factor for otitis media and pneumococcal infection in infants (50) is highly prevalent in France: the absence of breast-feeding beyond the first weeks of life. Breast-feeding practices vary considerably throughout Europe (51). A national survey conducted in 1995 among 12,179 babies at French maternity hospitals showed that France had the lowest level among Western countries for which national data on breast-feeding were available: only 52% of newborns were breastfed at hospital discharge, including 10% of babies partially breastfed (52). Efforts to encourage breast-feeding are needed in France to promote infant health and decrease susceptibility to respiratory tract infections.

### Differences in Regulatory Practices

Antibiotic prescriptions are affected through reimbursement policies and the structure of the pharmaceutical market. The average level of retail prices for pharmaceutical products is very low in France. For example, if the price in France is 100, the level would be 162 in the United Kingdom and 175 in Germany (53). Another study (54) demonstrated that Laspeyres (U.S. quantity-weighted) indexes for prices per drug dose show large differences compared to the reference country (USA): Germany, +24.7%; Canada, +2.1%; Japan, –12%; Italy, –13%; United Kingdom, –17%; and France, –32%. Because of this low level of pharmaceutical pricing, France not only is ranked first in the consumption per capita of outpatient antibiotics but also had the 3rd highest consumption of pharmaceutical products per capita among all countries in the Organization for Economic Cooperation and Development in 1997 (55). The overall per-capita expenditures on pharmaceuticals in 1997, adjusted for cost-of-living differences, were $352 in France versus $294 in Germany (56).

Historically, the French drug economy has largely been regulated by product price control and has been structured as a low-price, high-quantity system, whereas Germany has tended more towards a high-price, low-quantity system (53,57). The different systems of price regulation are responsible to some extent for three important features that influence antibiotic prescribing patterns. First, generic medicines have played only a minor role (<5%) in the French pharmaceutical market (58,59), but they account for 39% of all prescribed medicines in volume and 38% in value in Germany (59). This feature contributes to the observed trend in France of using newer antibiotics; in Germany, by contrast, narrow-spectrum, generic agents are more commonly used (19). A second factor is that until recently, French pharmacies were better remunerated if they dispensed large volumes of relatively expensive drugs such as oral broad-spectrum cephalosporins (59). By contrast, pharmacy remuneration in Germany is calculated by applying regressive percentages to different price bands: the lower the price, the higher the pharmacist’s share (60). Finally, the French pricing system has induced companies to develop aggressive promotional efforts and marketing campaigns to curb sales and compensate for low prices (53). Consequently, we speculate that French and German general practitioners are exposed to very different marketing information on antibiotics (12,29). However, representative data for both countries are not publicly available on that issue.

Most importantly, health authorities in Germany have more regulatory power by allocating collective expenditure caps and, therefore, have a broader impact on drug use than that exerted by similar agencies in France. In 1993, the introduction of capped physician budgets and a system of reference pricing in Germany led to a switch in prescribing preferences and an incentive for German physicians to avoid expensive products priced above the reference price, such as oral broad-spectrum cephalosporins (54). Consequently, from 1994 to 1997, the volume of antibiotics prescribed decreased temporarily from 334 million to 305 million DDDs (57).

In France, the introduction of national prescription guidelines (Références Médicales Opposables) for upper respiratory tract infections in 1994 did not decrease the overall volume of outpatient antibiotic use and had only a modest economic impact. However, prescription patterns have changed in line with those guidelines and led to a decrease in the use of fluoroquinolones and oral cephalosporins and to a substantial increase in macrolide use for acute bronchitis and pharyngitis (61).

### Possible Alternative Explanations

Several alternative explanations for the observed differences in antibiotic resistance rates can be made. First, variation in antibiotic use may be caused by differences in the frequency of respiratory tract infections. However, the rate of antibiotic consumption in France implies a rate of bacterial respiratory illness that is at least 5 times higher than the reported rates in the literature (20); therefore, the high antibiotic usage cannot be justified by known rates of the principal bacterial infections of the respiratory tract encountered in the community. As stated by Guillemot et al. (22), the observed increase in respiratory tract infections with a presumed viral cause cannot be explained by demographic evolution, age distribution, or by the occurrence of large epidemics in France. Second, clonal differences may be responsible for the observed differences. However, we did not identify any study suggesting that the circulating strains and serotypes of antibiotic-resistant pneumococci in France are intrinsically more virulent or transmissible compared to strains circulating in Germany (62,63). Third, another possible reason for the epidemiologic gap between both countries might be differences in diagnostic practice. For instance, two recent studies suggest underdiagnosis of invasive pneumococcal disease in Germany (64,65). However, we have no reason to believe that France has a significantly higher detection and identification rate of pneumococcal infection, when considering the previously mentioned diagnostic practices in France (22,23). Finally, although obtaining comparable data about the severity of illness of outpatients in France and Germany is difficult, no evidence shows that the French health-care system is more likely to treat patients who are more severely ill or who have a higher likelihood of severe infection (66). Nevertheless, the heterogeneity of patient populations and their varying susceptibility to infection should be better described in future studies about international differences in antibiotic use and resistance rates.

## Conclusions

This report represents a unique attempt to combine different data sources to give a more complete picture of sociocultural and economic forces influencing the ecology of antibiotic use and pneumococcal resistance in two large European countries. The published literature regarding the prevalence of antibiotic-resistant pneumococci provides convincing evidence that France and Germany have sharply different rate. The reasons for the observed resistance gap are multifactorial and include substantial differences in physicians’ and patients’ attitudes towards antibiotics; sociocultural and economic factors; and disparities in regulatory practices. Studies are remarkably consistent in documenting the high frequency with which antibiotics are used in France for upper respiratory tract infections without appropriate microbiologic rationale. Unfortunately, despite the widespread publication of recommendations over the last decade and some modest modifications in the pattern of antibiotic utilization (61), the willingness of French general practitioners to change their antibiotic-prescribing habits has been at best grudging and at worst nonexistent (29,67). As shown in a recently published survey from the United States (68), little evidence suggests that national guidelines alone, particularly when they emphasize societal concerns, have much impact on individual antibiotic-prescribing behavior. Therefore, much more attention needs to be focused on patient expectations and perceptions of illness and the constraints of medical practice (39). Major improvements are needed in communicating to individual patients (69) and in informing the general public about the risks of inappropriate antibiotic use (13,70). In November 2001, the former French Minister of Health, Dr. Bernard Kouchner, took an important step in this direction by allocating 30 million euros for public awareness campaigns about antibiotic misuse and resistance (71). In this respect, France may also learn from the experience of countries like the United States, Canada, Belgium, and Sweden, which all managed to reduce excessive antibiotic use on a national level (19,72–74). In particular, a number of Scandinavian studies have suggested that national antibiotic policies together with public information campaigns and changes in reimbursement policies can be effective (55,75).

In Germany, low prevalence of pneumococcal resistance coincides with less antibiotic consumption, selection of narrow-spectrum antibiotics, higher dosing of amino-penicillins, and possibly, better treatment compliance (2). Thus, learning from Germany’s experience with regard to the low prevalence of penicillin-resistant pneumococci may have some value for other countries. However, German health authorities should be careful in regard to the increasing spread of antibiotic-resistant pneumococci (11,76). A rational approach to the control of antibiotic-resistant pneumococci and the surveillance of antibiotic use in the outpatient setting are urgently warranted in Germany to preserve the still favorable situation.

An interesting question remains about whether differences in national antibiotic-prescribing patterns affect the rates of illness and death from complications of respiratory tract infections. A recent study (77) showed, for instance, that the Netherlands, a country with low antibiotic prescription rates for acute otitis media, had an incidence rate of acute mastoiditis of 3.8/100,000 person-years, whereas in countries with very high prescription rates, incidence rates were considerably lower, ranging from 1.2 to 2.0/100,000 person-years. A conservative approach and withholding of antibiotics in the treatment of acute otitis media may also have increased the occurrence of acute mastoiditis in Germany (78). A recently published article (79) indirectly suggests that the low rates of *Haemophilus influenzae* type B meningitis in some countries, particularly in Asia, may be due at least partially to extensive antibiotic use. However, no recently published, representative surveillance data exist about illness and death from complications of common respiratory tract infectious in France or Germany. Future international studies about the use of outpatient antibiotics (80) should include cross-country surveillance data for serious infectious complications such as mastoiditis, acute rheumatic fever, meningitis, or suppurative complications of pharyngitis (81).

Finally, we argue that effects exerted at the macro-level by the cultural and socioeconomic environment contribute substantially to the observed differences in prescribing practices and related antibiotic resistance rates. Consequently, failure to understand the sociocultural and economic perspectives of antibiotic consumption and resistance will lead to inadequate conclusions about the chances of success for possible interventions. More research to inform decision-makers on the determinants of the variation in antibiotic use and resistance patterns is urgently needed.
